# The Role of Nanoparticle Shapes and Structures in Material Characterisation of Polyvinyl Alcohol (PVA) Bionanocomposite Films

**DOI:** 10.3390/polym12020264

**Published:** 2020-01-25

**Authors:** Mohanad Mousa, Yu Dong

**Affiliations:** 1 School of Civil and Mechanical Engineering, Curtin University, GPO Box U1987, Perth 6845, Australia; mohanadmousa616@yahoo.com; 2 Shatrah Technical Institute, Southern Technical University, Basra 61001, Iraq

**Keywords:** polyvinyl alcohol (PVA), bionanocomposites, nanomechanical behaviour, thin films

## Abstract

Three different types of nanoparticles, 1D Cloisite 30B clay nanoplatelets, 2D halloysite nanotubes (HNTs), and 3D nanobamboo charcoals (NBCs) were employed to investigate the impact of nanoparticle shapes and structures on the material performance of polyvinyl alcohol (PVA) bionanocomposite films in terms of their mechanical and thermal properties, morphological structures, and nanomechanical behaviour. The overall results revealed the superior reinforcement efficiency of NBCs to Cloisite 30B clays and HNTs, owing to their typical porous structures to actively interact with PVA matrices in the combined formation of strong mechanical and hydrogen bondings. Three-dimensional NBCs also achieved better nanoparticle dispersibility when compared with 1D Cloisite 30B clays and 2D HNTs along with higher thermal stability, which was attributed to their larger interfacial regions when characterised for the nanomechanical behaviour of corresponding bionanocomposite films. Our study offers an insightful guidance to the appropriate selection of nanoparticles as effective reinforcements and the further sophisticated design of bionanocomposite materials.

## 1. Introduction

Nanoparticles in spheroidal, platelet-like, and tubular shapes as effective nanofillers have attracted materials engineers and researchers in the field of nanocomposite materials in the past few decades [1,2]. The incorporation of different nanoparticles into continuous polymer matrices has been proven to significantly alter the properties of virgin polymers, resulting in a novel-class system of polymer nanocomposites with superior properties and excellent functionalities [1,2]. In general, when embedded with a small fraction of nanoparticles being less than 10 wt %, the optical [3], mechanical [4], thermal [5], electronic [6], and antimicrobial [7] properties of polymer nanocomposites can be remarkably enhanced while maintaining some features of net polymer systems such as low density and easy processibility [2]. Such polymer nanocomposites possess a wide range of applications including medical devices, aerospace engineering, and automotive components [1]. For instance, nanocomposites reinforced with some polymeric and inorganic nanofillers such as chitosan nanoparticles and silver nanoparticles have been proven to be effective for antimicrobial treatment in dentistry [8] or for bone tissue regeneration [9]. Other studies [10,11] demonstrated that polymer nanocomposites, as exemplified by polypropylene (PP)/clay nanocomposites, have real automotive industrial potential to result in significant property improvement with only minor increasing cost if a deeper understanding of their structure–property relationship can be achieved.

The effective reinforcing mechanism is based on the fundamental concept that the chain mobility of polymeric molecules is restricted by rigid nanofillers according to the matrix–particle interfacial interactions in polymer nanocomposites [2,12]. The specific areas associated with matrix–filler interactions are known as interfacial regions with completely distinct properties from those of nanoparticles and polymer matrices individually. More importantly, the material performance of polymer nanocomposites primarily depends on the volume of interfacial regions and interfacial properties [12] in relation to critical nanofiller parameters such as nanoparticle shapes and structures.

In addition to a major concern of nanoparticle structures, nanoparticle shapes are also equally important when matrix–filler interaction is considered in polymer nanocomposites, which can be classified into three popular shapes, namely 1D platelet-like nanoparticles such as montmorillonite (MMT) clays and nanoplatelet graphene sheets, 2D tubular nanoparticles such as HNTs and carbon nanotubes (CNTs), as well as 3D spherical nanoparticles such as diamond nanoparticles and nanosilica particles and fractal-like or irregular near circular-like nanoparticles such as NBCs. In a nanocomposite system, the alteration of nanoparticle shapes means that the contact areas inevitably vary between polymer matrices and nanoparticles to effectively control the volume of their interfacial regions [12]. Most previous studies [13,14] were based on theoretical or numerical modelling approaches such as atomistic and coarse-grained molecular dynamic (MD) simulations for evaluating the matrix–filler bonding effect. Nonetheless, current computational capability and the environment may be mostly restricted to the context of single and two-particle systems by neglecting the effect of actual nanoparticle structures and shapes induced in different material processing techniques [15].

The main objective and novelty of this study lie in holistically assessing the influence of different dimensional nanoparticle shapes, structures, and contents on the effective reinforcement mechanism of PVA bionanocomposites reinforced with 1D Cloisite 30B clays, 2D HNTs, and 3D NBCs, respectively. The selection of PVA as a base polymer arises from its good biodegradability and water solubility to replace conventional petroleum-based polymers for generating much less marine plastic wastes [16]. Our study demonstrated that 3D NBCs could act as relatively new and superior carbon-based nanofillers to clay-based Cloisite 30B and HNTs for the best material performance of nanocomposites. This highlighted their great benefit to be more competitive nanoreinforcements in the manufacture of composite materials, as well as future potential to electronics, material packaging, and biomedical applications.

## 2. Materials and Methods

### 2.1. Materials

PVA (material type: MFCD00081922), as a popular water-soluble biopolymer, was purchased from Sigma Aldrich Pty. Ltd., Castle Hill, NSW, Australia with the molecular weight of 89,000–98,000 g/mol and the degree of hydrolysis of 99.0%–99.8%. Three different types of nanoparticles used in this study comprised Cloisite 30B clays, HNTs, and NBCs. In between, NBCs were directly purchased from US Research Nanomaterials, Inc. Co., Houston, TX, USA (molecular weight: 12.01 g/mol, particle density: 0.43 g/cm^3^, and particle size <69.43 nm [4]). Moreover, Cloisite 30B clays with methyl, tallow, bis-2-hydroxyethyl, quaternary ammonium were supplied by Southern Clay Products, Gonzales, LA, USA while HNT powders, donated by Imerys Tableware Limited, Auckland, New Zealand, have particle dimensions of 120–140 nm in outer dimeter, 15–100 nm in inner diameter, and 0.3–1.5 µm in length [17].

### 2.2. Fabrication of PVA Bionanocomposite Films

All PVA bionanocomposite films reinforced with Cloisite 30B clays, HNTs, and NBCs were prepared using solution casting according to the fabrication procedure mentioned in our previous work [4]. Initially, 5 wt %/v PVA aqueous solution was prepared by mixing 10 g PVA into 190 mL deionised water under vigorous magnetic stirring at 400 rpm and 90 °C for 3 h until PVA was completely dissolved. Aqueous suspensions of all nanoparticles were achieved by mechanical mixing filler powders in deionised water with a rotor speed of 405 rpm at 40 °C for 2 h, which was followed by the ultrasonication (Model ELMA Ti–H–5, Elma Schmidbauer GmbH, Singen, Germany) at 25 kHz and 40 °C with a power intensity of 70% for 1 h. Subsequently, nanoparticle contents of 0, 3, 5 and 10 wt % were obtained by controlling PVA amounts used in each material formulation. Then, such aqueous suspensions were gradually added in a dropwise manner into PVA solutions and simultaneously subjected to mechanical mixing at 405 rpm and 40 °C for 2 h. Afterwards, their mixtures were stirred at 400 rpm and 90 °C for 1 h prior to the sonication for 30 min to achieve uniform nanoparticle dispersion. Finally, 20 mL prepared solution was poured on a glass Petri dish and allowed to dry in an air-circulating oven at 40 °C for 48 h. Subsequently, different types of PVA bionanocomposite films were stored in a silica gel-containing desiccator prior to material testing.

### 2.3. Characterisation Methods

In this study, nanomechanical properties of PVA bionanocomposite films were quantitatively assessed in peak force quantitative nanomechanical mapping (PFQNM) [12] via atomic force microscopy (AFM) based on a Bruker Dimension Fastscan AFM system (Bruker Corporation, Karlsruhe, Germany). A Tapping Mode Etched Silicon Probe (TESPA) was employed with the nominal spring constant of 40 N/m and tip radius of 8 nm. The image scan rate was controlled at 2 Hz with 256 × 256 digital pixel resolution. In order to remove unwanted noise, bow and tilt features from the vertical scanner (*Z*) and AFM topographic images were first-order flattened via the *Flatten* command with the aid of Burker Nanoscope 1.5 software (Bruker Corporation, Karlsruhe, Germany).

Fourier transform infrared (FTIR) spectrometry (PerkinElmer Spectrum 100 FTIR spectrometer, PerkinElmer, Waltham, MA, USA) was utilised to characterise the chemical bonding effects of PVA, nanoparticles as well as PVA bionanocomposites in a wavenumber range of 650–4000 cm^−1^ with a resolution of 4 cm^−1^ according to an attenuated total reflectance (ATR) method.

Additionally, X-ray diffraction (XRD) analysis was carried out using a Bruker D8 Advanced Diffractometer (Bruker Corporation, Karlsruhe, Germany). The X-ray source was Ni-filtered Cu-Kα radiation (wavelength λ = 0.1541 nm) carried out at the accelerating voltage and current of 40 kV and 40 mA, respectively. X-ray spectra were recorded in a 2*θ* range of 10–50° at the scan rate of 0.015°/s.

A universal testing machine (Lloyd EZ50, Lloyd Instruments Ltd., West Sussex, UK) was employed at room temperature at the crosshead speed of 10 mm/min (gauge length: 50 mm) in order to measure the tensile properties of neat PVA and PVA bionanocomposite films according to ASTM D882-02. For each material batch, six specimens were tested with the mean values and standard deviations being calculated accordingly. Moreover, the tensile toughness was also determined based on tensile energy to break (TEB) with reference to ASTM D882-02.

The fracture morphology for tensile testing specimens was evaluated with the aid of a field emission scanning electron microscope (FE-SEM, Zeiss NEON 40 EsB Cross Beam, Carl Zeiss Microscopy GmbH, Jena, Germany) at an accelerating voltage of 5 kV after being coated with platinum (layer thickness: 5 nm).

The thermal properties of PVA bionanocomposite films were examined using a combined measurement system based on thermal gravimetric analysis (TGA) and differential scanning calorimetry (DSC) (1 STAR^e^ system, Mettler-Toledo, Columbus, OH, USA) from 35 to 700 °C at a scan rate of 10 °C/min and a flow rate of 25 mL/min under argon atmosphere. The degree of crystallinity *χ_c_* of PVA matrices within PVA bionanocomposites was calculated as follows:

(1) $$\chi_{C}\;\left(\%\right)=\frac{\Delta H_{m}}{w\Delta H_{m}^{O}}\times 100\%$$

where $\Delta H_{m}$ is the measured melting enthalpy according to DSC data. $\Delta H_{m}^{O}$ = 138.6 J/g [5] is the enthalpy of fully crystalline PVA, and *w* is the weight fraction of PVA matrices in corresponding PVA bionanocomposites.

## 3. Results and Discussion

### 3.1. Nanoparticle Shape and Size

**Figure 1:**
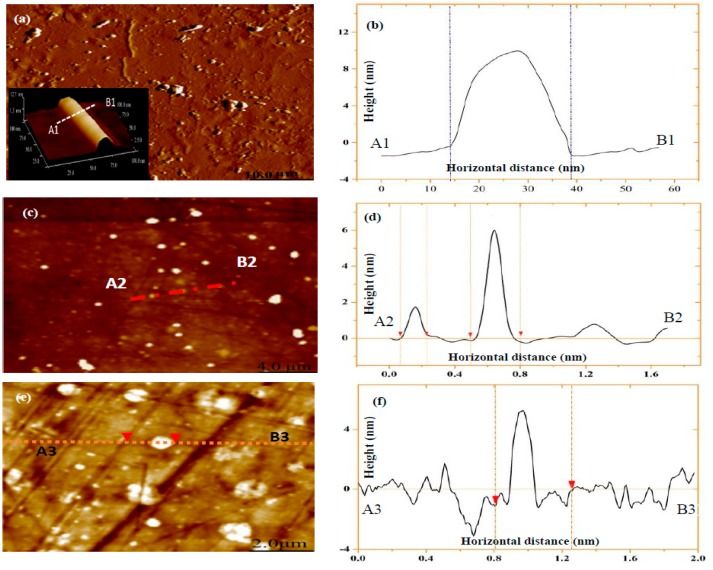
Atomic force microscopy (AFM) characterisation of different nanoparticles: AFM images of (**a**) HNTs, (**c**) Cloisite 30B clays, and (**e**) NBCs deposited on mica substrate in aqueous solutions, and height profiles of (**b**) HNTs, (**d**) Cloisite 30B clays, and (**f**) NBCs at typical cut sections A1–B1, A2–B2, and A3–B3, respectively.

The morphological structures of as-received nanoparticles of Cloisite 30B clays, HNTs, and NBCs are illustrated in Figure 1. All nanoparticles powders show high irregularities in size and material morphology. However, HNTs are most likely to possess cylindrical shapes with transparent central areas running longitudinally along such cylindrical structures, as illustrated in Figure 1a,b. The outer diameters and lumen diameters of HNTs, as typical tubular nanoparticles in hollow and open-end structures, were found to be in range of 20–115 and 5–30 nm, respectively. Whereas, the lengths of HNTs vary from 50 nm to 1.5 µm. On the other hand, the morphological structures of Cloisite 30B clays were detected using an AFM tapping mode from diluted clay suspension when deposited onto the mica substrate, as revealed in Figure 1c,d. It is evident that Cloisite 30B clays possess platelet-like structures with an average particle diameter of approximately 100.75 ± 6.5 nm by measuring 925 clay particles, while their thickness varies from 1.69 to 5.9 nm, which is exemplified by a cross-sectional analysis for a typical section (A2–B2) illustrated in Figure 1d. These results suggest that clay platelet-like layered structures can consist of single platelets (thickness: ~1 nm) [18] as well as stacked layered platelets, signifying the combination of clay exfoliation and intercalation. NBC sizes were determined previously to be 69.43 nm by average [4], as shown in Figure 1e,f, which can be considered as 3D anisotropic nanoparticles as opposed to 1D platelet-like clays and 2D HNTs.

### 3.2. Chemical Bonding Effect

**Figure 2:**
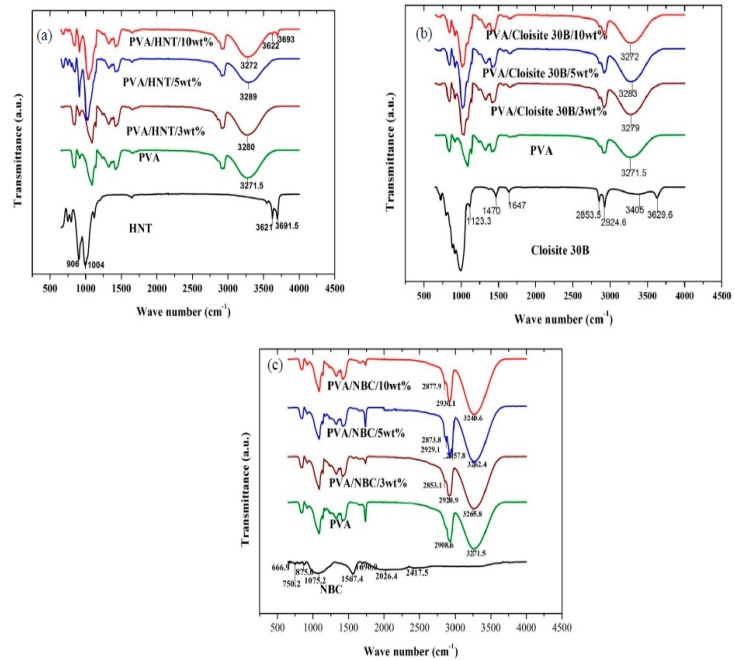
FTIR spectra for chemical interactions of polyvinyl alcohol (PVA) bionanocomposite films reinforced with (**a**) HNTs, (**b**) Cloisite 30B clays, and (**c**) NBCs.

FTIR was employed to evaluate functional groups in PVA matrices and nanoparticles as well as their associated chemical bonding effects. As-received HNTs exhibit two Al_2_OH stretching bands assigned to 3691.5 and 3621 cm^−1^ in Figure 2a, resulting from OH bending in connection with two Al atoms along with other band features of inorganic aluminosilicate structures of halloysite [19]. Furthermore, FTIR peaks observed at 1004 and 906 cm^−1^ are associated with Si–O–Si and Al–OH stretchings, respectively. In comparison, as-received Cloisite 30B clays have an existing peak at 3629.6 cm^−1^ corresponding to Si–OH and Al–OH stretchings in Figure 2b. The broad band at 3405 cm^−1^ is assigned to OH groups in relation to interlayer water, while two typical bands at 2924.6 and 2853.5 cm^−1^ are related to –CH_2_ asymmetric and symmetric stretchings, respectively [20,21]. Moreover, FTIR peaks at 1647 and 1123.3 cm^−1^ can be due to the deformation vibration of interlayer water and Si–O bending accordingly, as opposed to an assigned band at 1470 cm^−1^ arising from –CH_2_ bending [20]. The NBC results presented in Figure 2c confirm the absence of –OH groups in their FTIR spectra, which infers much lower moisture and alcohol contents obtained in NBCs. Additionally, FTIR peaks at 2417.5 and 1567.4 m^−1^ reveal the existence of C≡H stretching [22] and C=C vibration in an aromatic system [23], respectively. On the other hand, the peak spectrum at 1696 cm^−1^ was assigned to the C=O band primarily for ionisable carboxyl groups as an indicator of surface hydrophilicity [24].

In the case of bionanocomposite systems, the FTIR spectra of PVA/HNT bionancomposites and PVA/Cloisite 30B clay bionanocomposites are also illustrated in Figure 2a,b, respectively. The FTIR peak located at 3271.5 cm^−1^ associated with O–H stretching shifts to higher wavenumbers at 3280 and 3289 cm^−1^, as well as 3279 and 3283 cm^−1^ with the inclusion of HNTs and Cloisite 30B clays at the nanoparticle contents of 3 and 5 wt %, respectively. Such a finding was attributed to the strengthening effect of hydrogen bonds between –OH groups from PVA molecules and those located on clay surfaces such as silanol groups (–SiOH), which is in good agreement with previous investigations on PVA/organomodified Cloisite Na^+^ (OMMT) nanocomposites [21], poly (ε-caprolactone) (PCL)/Cloisite 30B clay nanocomposites [25], and PVA/chitosan (CS)/HNT nanocomposites [26]. However, when the HNT content increases up to 10 wt %, two Al_2_OH stretchings appear for embedded HNTs in bionanocomposite films due to typical HNT agglomeration [27]. As for PVA/NBC bionancomposite films, increasing the NBC content from 0 to 10 wt % leads to the band-peak shift to a lower wavenumber at 3240.6 cm^−1^ owing to large amounts of hydroxyl groups in PVA molecules [4] as well as strong filler–matrix bonding. As a result, hydrogen bonds are generated to be intertwined at PVA/NBC interfaces with a broad O–H band. Such a variation associated with –OH stretching vibration proves the formation of hydrogen bonds, which is in good accordance in PVA/graphene nanocomposites [27] and PVA/bamboo charcoal (BC) nanocomposites [5]. The aforementioned results fail to show existing new bands in PVA films with the inclusion of both HNTs and Cloisite 30B clays. On the contrary, the addition of 3 and 5 wt % NBCs within PVA matrices in bionanocomposite films gives rise to a new band in relation to –CH_2_-asymmetric and symmetric stretchings [4]. Such a finding can be ascribed to typical NBC porous structures enabling absorbing molecular chains of hydrophilic polymers such as PVA with the combined mechanical and chemical bondings. More consistently, the incorporation of HNTs and Cloisite 30B clays in PVA bionanocomposites shifts the hydroxyl peaks of PVA to relatively high wavenumbers compared to the addition of NBCs [4]. Such results indicate that the numbers of hydrogen bonding generated in PVA/HNT bionanocomposites and PVA/Cloisite 30B clay bionanocomposites are higher when compared with those detected in PVA/NBC bionanocomposites, owing to different chemical structures of nanofillers. Since NBCs do not possess –OH peaks, most hydrogen bonding formed in bionanocomposites can arise from hydroxyl groups of PVA molecules. Whereas, existing –OH peaks detected in HNTs and Cloisite 30B clays in PVA bionanocomposites are believed to further facilitate the formation of more hydrogen bonds within PVA matrices.

### 3.3. XRD Patterns

**Figure 3:**
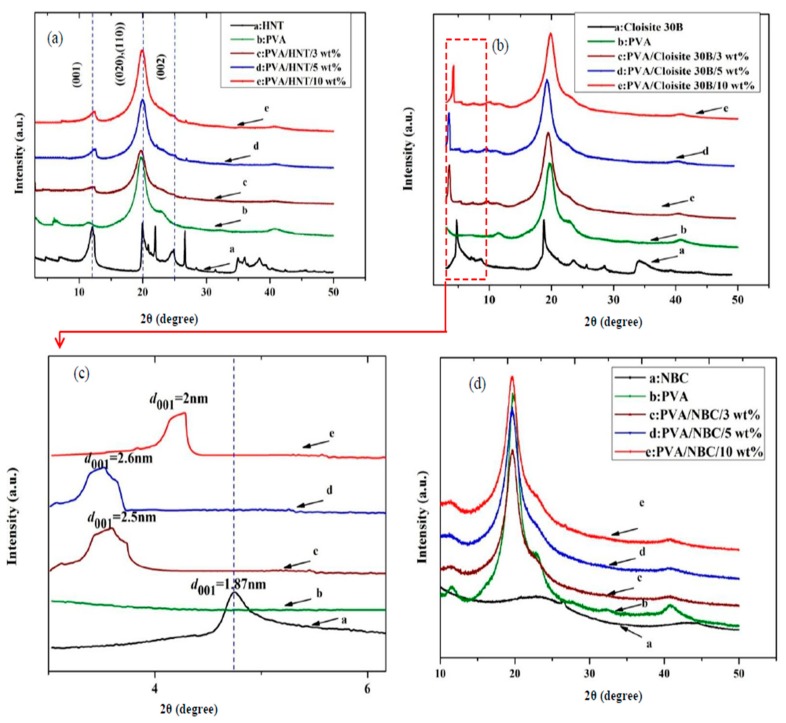
XRD patterns for PVA bionanocomposites reinforced with (**a**) HNTs, (**b,c**) Cloisite 30B clays with both wide and small diffraction angles, respectively, and (**d**) NBCs.

XRD analysis is a very useful material characterisation method to evaluate the crystalline structures of polymers and composites as well as to determine *d*-spacing values between clay interlayers. By monitoring the position and intensity of basal reflections from distributed silicate layers, nanocomposite structures (i.e., intercalated or exfoliated) as well as clay aggregated structures can be identified accordingly [28,29]. The XRD patterns of HNTs and corresponding nanocomposites are presented in Figure 3a. HNT patterns possess three major peaks of (001), (020)/(110), and (002) located at 2*θ* = 11.9°, 20°, and 24.9°, leading to *d*-spacing values of 0.74, 0.44, and 0.37 nm, respectively. The relevant peak taking place at 2*θ* = 24.9° is attributed to the presence of silica in the form of cristobalite and quartz [30]. After the incorporation of HNTs into PVA matrices in nanocomposite systems, the XRD characteristic peak at 2*θ* = 11.9° appears to be very weak at the low HNT content of 3 wt %. Similar XRD peaks have been detected at 2*θ* = 12.5° and 12.6° with slight peak shift at relatively high HNT contents of 5 and 10 wt %, respectively. Overall, decreasing the HNT content significantly reduces the intensity of XRD peaks for all PVA/HNT nanocomposites, which may be due to uniform HNT dispersion at the low HNT content levels. Such a phenomenon suggests that the disappearance or intensity reduction of XRD peaks at low HNT contents results from uniform HNT dispersion in a more randomly oriented manner. On the other hand, the reappearance of XRD peaks at higher HNT contents is indicative of possible HNT agglomeration.

The XRD patterns of Cloisite 30B clays reveal the diffraction peak at 2θ=4.72° corresponding to the *d*-spacing value of 1.87 nm, as shown in Figure 3b,c. The (001) diffraction peak shifted to lower angles, as evidenced by the *d*-spacing values of 2.5, 2.6, and 2 nm for PVA/Cloisite 30B nanocomposites at the clay contents of 3, 5 and 10 wt %, respectively. This phenomenon clearly arises from the diffusion of polymeric chains inside clay interlayers to induce clay intercalation in agreement with PVA/Na^+^ MMT nanocomposites [21] and PLA/Cloisite 30B nanocomposites [31]. The XRD peak for PVA alone appears at 2θ=19.7°, which is associated with the total (101) crystalline atactic formation of PVA molecular chains [32] to slightly shift to lower diffraction angles when increasing the clay content in PVA bionanocomposites. The occurrence of PVA molecular chains at the (101) crystalline plane suggests that PVA matrices evolve towards crystalline structures under more constraints. A similar behaviour was also reported in PVA/clay nanocomposites [32,33], which is ascribed to the strong chemical interactions between nanofillers and polymer matrices. The aforementioned results indicate that Cloisite 30B clays are successfully intercalated and/or exfoliated by PVA molecular chains, and HNTs are homogenously dispersed at their low contents within continuous PVA matrices, which is attributed to active interactions between PVA matrices and clay nanoparticles due to strong hydrogen bonding taking place between carboxyl groups of PVA molecules and hydroxyl groups in the interlayer areas of Cloisite 30B clays or on the surface edges of HNTs [28].

In comparison, the XRD patterns of NBCs demonstrate two broad XRD peaks, as depicted in Figure 3d. The broad peaks located at 2*θ* = 22.9° are associated with the sharp peaks of graphite assigned to the (002) diffraction plane [34]. Besides, the second broad peak detected at 2θ=43.6° characterises 2D in-plane symmetry (101) along with graphene layers. Moreover, XRD patterns of PVA/NBC bionanocomposites only show the diffraction angles from PVA, as illustrated in Figure 3d, which is consistent with the previous finding [35] in PVA/5 wt % graphene oxide (GO) nanocomposites with a clear disappearance sign of GO diffraction peaks in regular and periodic structures, leading to individually exfoliated GOs in PVA matrices.

### 3.4. Topographic Surface Morphology and Roughness

**Figure 4:**
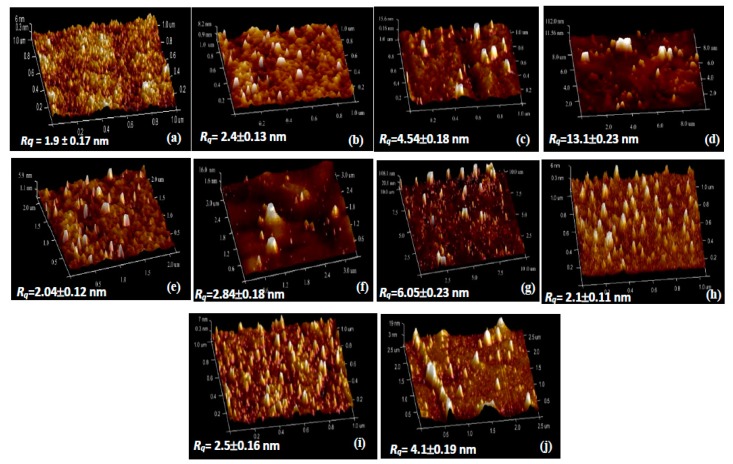
3D AFM height mapping images of (**a**) PVA and PVA bionanocomposites reinforced with (**b**) 3 wt % HNTs, (**c**) 5 wt % HNTs, (**d**) 10 wt % HNTs, (**e**) 3 wt % Cloisite 30B clays, (**f**) 5 wt % Cloisite 30B clays, (**g**) 10 wt % Cloisite 30B clays, (**h**) 3 wt % NBCs, (**i**) 5 wt % NBCs, and (**j**) 10 wt % NBCs.

To assess nanofiller dispersion within PVA matrices, 3D height mapping images of PVA and PVA bionanocomposites are exhibited in Figure 4. As illustrated in Figure 4b, HNT nanoparticles are separated from one another with better HNT dispersion in PVA bionanocomposites reinforced with 3 wt % HNTs, as opposed to typical clay agglomeration and clustering issues beyond the 3 wt % HNTs shown in Figure 4c,d. An excessive amount of HNTs results in decreasing intraparticle spacing along with the higher intramolecular bonding of HNTs, leading to particle agglomeration [36]. Besides, the average root mean square (*R_q_*) value as an indicator of the surface roughness of PVA bionanocomposites has been reported to be 2.4 ± 0.13 nm at the HNT content of 3 wt % when compared with 1.9 ± 0.17 nm for neat PVA, as shown in Figure 4a. It is suggested that the smooth surfaces of PVA/HNT bionanocomposites remain with the incorporation of HNTs at relatively low HNT contents, owing to their uniform dispersion. Such a finding is consistent with PVA/HNT hydrogels mentioned elsewhere [37]. At the low HNT content of 3 wt %, the smooth surfaces of PVA/HNT bionanocomposites can also be ascribed to the combination of strong interactions and good compatibility between HNTs and PVA matrices. Nonetheless, increasing the HNT content up to 5 and 10 wt % leads to much higher surface roughness (i.e., *R_q_* = 4.54 ± 0.18 and 13.1 ± 0.23 nm, respectively) due to the presence of prevalent HNT aggregates [38].

On the other hand, PVA/Cloisite 30B clay bionanocomposites and PVA/NBC bionanocomposites reveal different dispersibilities as opposed to PVA/HNT bionanocomposites. When nanofiller contents are below 10 wt %, spiky nanoparticles appear to be separated from one another, resulting in the homogeneous dipsersion of Cloisite 30B clays and NBCs within PVA matrices, as shown in Figure 4e,f, as well as Figure 4h,i accordingly. In particular, as the nanofiller content increases from 3 to 5 wt %, *R_q_* values increase moderately from 2.04 ± 0.12 to 2.84 ± 0.18 nm for PVA/Cloisite 30B clay bionanocomposites, as well as from 2.1 ± 0.11 to 2.5 ± 0.16 nm for PVA/NBC bionanocomposite in contrast with 1.9 ± 0.17 nm for neat PVA. This finding suggests that the smooth surfaces for PVA bionanocomposites are evident at the low nanofiller contents of Cloisite 30B clays and NBCs, which is in good agreement with previous studies of PVA/nanocelloluse composite films [39]. On the contrary, the inclusion of 10 wt % Cloisite 30B clays and NBCs in PVA bionanocomposites consistently gives rise to increasing *Rq* values up to 6.05 ± 0.23 and 4.1 ± 0.19 nm, respectively, which are also far higher than that of neat PVA at 1.9 ± 0.17 nm. Such results indicate that the presence of aggregated Cloisite 30B clays and NBCs results in a much higher surface roughness on PVA surfaces, as expected. In comparison, the *R_q_* value of 4.1 ± 0.19 nm for PVA/NBC bionanocomposites appears to be relatively low as compared with those of other PVA nanocomposites reinforced with carbon-based fillers such as PVA/reduced graphene oxide (rGO) nanocomposites with a *R_q_* value of 4.6 ± 0.55 nm based on deposition layers [40].

**Figure 5:**
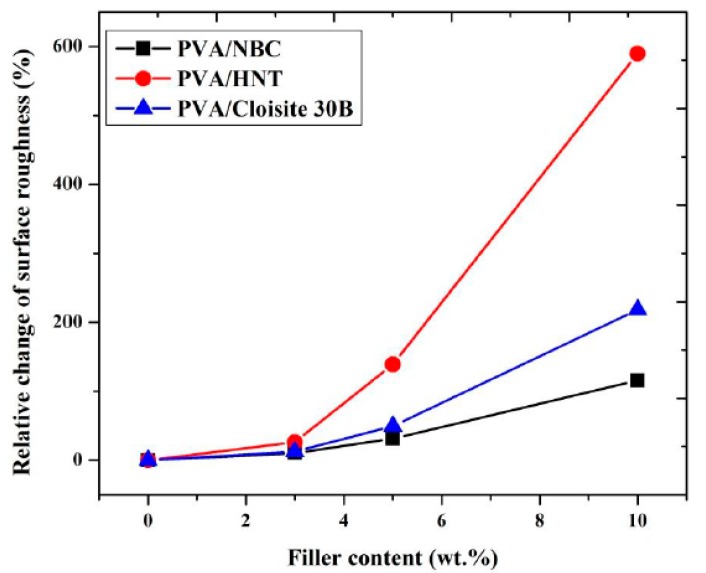
Relative change of surface roughness in terms of filler content in different PVA bionanocomposites.

Notwithstanding that the same manufacturing process condition and nanofiller contents have been utilised for preparing PVA bionanocomposite films, different nanoparticle types play an important role in changing the degree of surface roughness. Overall, with increasing the nanofiller content, the surface roughness of PVA bionanocomposites in this study is enhanced to a different extent, as evidenced by increasing the maximum relative change of surface roughness up to 589.4%, 218.4%, and 115.8%, respectively with the inclusion of HNTs, Cloisite 30B clays, and NBCs at the same filler content of 10 wt % shown in Figure 5. Such results suggest that NBCs may have better ability to be dispersed uniformly in PVA matrices as opposed to HNTs and Cloisite 30B clays due to their least increasing level in surface roughness, especially when beyond 5 wt % in filler content. Whereas, the effect of different nanoparticle shapes and sizes on the surface roughness of PVA bionancomposites becomes less pronounced at low filler contents below 3 wt %.

### 3.5. Aspect Ratios of Embedded Nanofillers in PVA Bionanocomposites

The aspect ratio of nanofillers is regarded as one of key factors in reinforcement efficiency and mechanical performance of nanocomposites, which is generally defined as the ratio between the largest dimensions over the smallest dimension of nanofillers. According to this fundamental concept, the largest dimension of nanofillers can be represented by the lengths of tubular HNTs and platelet-like Cloisite 30B clays or the diameters of NBCs, while the smallest dimension is represented by the diameter of HNTs or thickness of Cloisite 30B clays and NBCs [28].

For instance, it is well known that when nanoclays are uniformly dispersed within polymer matrices, the formation of their exfoliated or intercalated structures leads to the improvement of the mechanical performance of nanocomposites to different extent, which is totally different from agglomerated nanoclays, resulting in the deterioration of their mechanical properties [1].

To investigate the degree of clay-exfoliated structures in detail, the height profiles of clay platelets relative to those of PVA matrices have been determined (Figure S1 in the Supporting Information). The thickness of 3 wt % Cloisite 30B clays within PVA matrices in bionanocomposites appears to be in range of 0.85–1.43 nm, suggesting typical exfoliated clay structures in dispersion. MMT clays are well known to be exfoliated when their thickness is similar to that of individual clay platelets (i.e., ~1 nm) [18]. Gaume et al. [33] and other groups [41,42] also detected intercalated and exfoliated structures of MMT clays in the thickness range of 1.3–5 nm.

**Figure 6:**
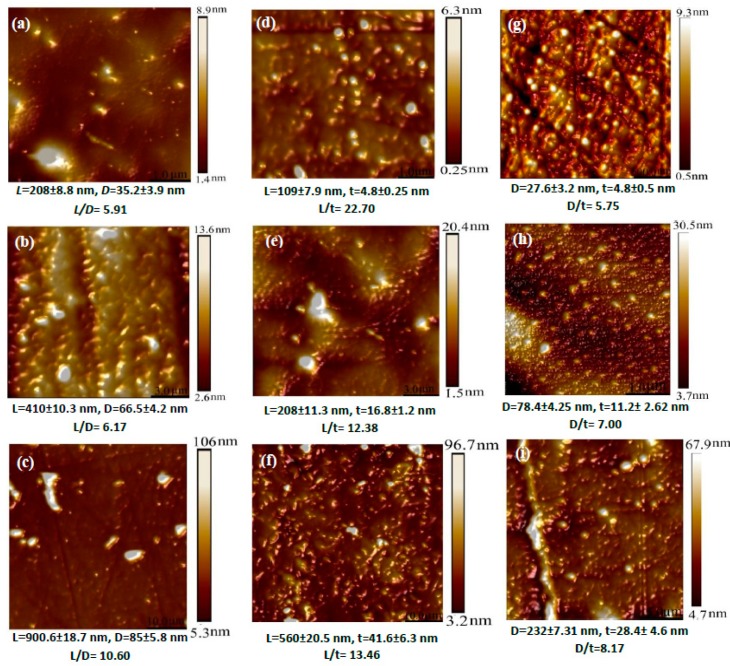
AFM topographic images of PVA bionanocomposites associated with aspect ratios of embedded fillers: (**a**) 3 wt % HNTs, (**b**), 5 wt % HNTs, (**c**) 10 wt % HNTs, (**d**) 3 wt % Cloisite 30B clays (**e**) 5 wt % Cloisite 30B clays, (**f**) 10 wt % Cloisite 30B clays, (**g**) 3 wt % NBCs (**h**) 5 wt % NBCs, and (**i**) 10 wt % NBCs.

The surface roughness mentioned earlier can be associated with nanofiller shapes and sizes since HNTs and Cloisite 30B clays may possess relatively high aspect ratios when compared with those of NBCs despite an existing ‘nanofiller waviness’ issue. HNTs and Cloisite 30B clays with high aspect ratios inevitably undergo considerable wavy nanofiller formation, thus undermining their homogeneous dispersion within polymer matrices [28]. Moreover, ultrasonication, as an effective fine nanoparticle dispersion technique in this study, also enables potentially damaging nanofiller structures particularly by applying high-power intensity or using a longer sonication time [43]. As such, the specific sizes/dimensions of nanofillers may vary to different extent, which are required to be determined for embedded HNTs, Cloisite 30B clays, and NBCs in PVA bionanocomposites to calculate their actual aspect ratios, as evidenced in Figure 6. The relevant frequency distribution of nanofiller dimensions are presented in Figures S2–S4 in Supporting Information as the reference. It is clearly revealed that the aspect ratios of nanofillers increase from 5.91 to 10.60 for HNTs in Figure 6a–c, as well as 5.75 to 8.17 for NBCs in Figure 6g–i with increasing the nanofiller content from 3 to 10 wt %. In contrast, the aspect ratios of Cloisite 30B clays decrease from 22.70, 12.38 to 13.46 when increasing the clay contents from 3, 5 to 10 wt % accordingly, despite their overall highest aspect ratios among all the nanofillers, as shown in Figure 6d–f. Such findings imply that the majority of Cloisite 30B clays tend to form exfoliated or intercalated clay structures with relatively high aspect ratios. However, the decrease in the aspect ratios of Cloisite 30B clays can be associated with more severe clay aggregation. It is very convincing that the aspect ratios of nanofillers can be greatly influenced by nanofiller shapes, and apparently 3D NBC nanoparticles have relatively low aspect ratios when compared with 1D platelet-like Cloisite 30B clays [28].

Overall, the aspect ratio may play a significant role in mechanical performance of nanocomposites when nanofiller shapes or structures are only considered within polymer matrices. However, for different types of nanofillers, several other factors such as the number of particles per unit volume, interphase modulus, interphase volume, and surface area, as well as the ratio of interphase volume per nanoparticle volume should also be taken into account for their overall material properties.

### 3.6. Mechanical Properties

**Figure 7:**
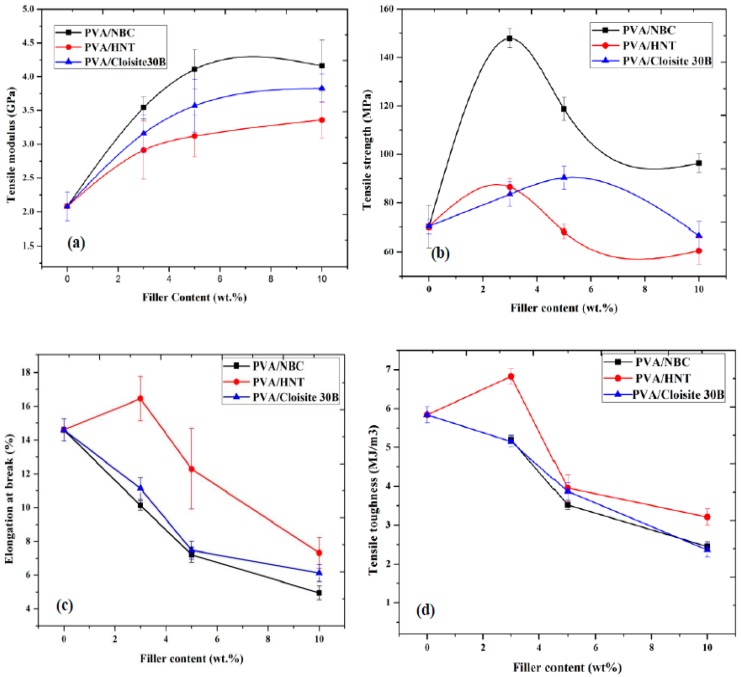
Mechanical properties of PVA bionanocomposites at different filler contents: (**a**) tensile modulus. (**b**) tensile strength, (**c**) elongation at break, and (**d**) tensile toughness.

Figure 7 displayed the mechanical properties of PVA bionanocomposites reinforced with HNTs, Cloisite 30B clays, and NBCs at different nanofiller contents. Overall, the tensile moduli of such bionanocomposites increase significantly in a monotonic manner with the increasing nanofiller content, as shown in Figure 7a. The addition of only 3 wt % HNTs, Cloisite 30B clays, and NBCs result in increasing the tensile moduli by 40%, 52%, and 70.67% as opposed to that of near PVA at 2.08 GPa, which is in good accordance with the previous results obtained in PVA/starch/ glycerol (GL)/HNT nanocomposites [44] and PVA/chitosan/HNT nanocomposites [45]. More remarkably, the maximum increases in tensile modulus are achieved by 61.5%, 84.1%, and 123% with the addition of 10 wt % HNTs, Cloisite 30B clays, and NBCs, respectively when compared with that of neat PVA. This phenomenon usually takes place for most polymers filled with more rigid inorganic nanoparticles, as the reinforcements lead to much stiffer nanocomposite materials [46]. In particular, NBCs induce more reinforcement efficiency as nanofillers when compared with Cloisite 30B clays and HNTs. This can be clearly seen from the overall relatively high tensile moduli of PVA/NBC bionanocomposites, arising from much closer interactions between PVA matrices and NBCs via the formation of mechanical and hydrogen bonds owing to highly porous structures of NBCs. On the other hand, a different trend for the tensile strengths of PVA/NBC bionanocomposites is clearly revealed from those of PVA/Cloisite 30B clay bionanocomposites and PVA/HNT bionanocomposites, as shown in Figure 7b. The tensile strength of PVA/HNT nanocomposites is improved by 23% with the addition of 3 wt % HNTs relative to that of neat PVA at 70.32 MPa. Nonetheless, a drastic strength-decreasing tendency takes place with the strength reductions of 3.2% and 13.9% when embedded with 5 and 10 wt % HNTs accordingly. Such results indicate that the enhancement of tensile strengths for PVA/HNT nanocomposites depends on effective stress transfer from PVA matrices to HNTs, resulting from homogeneous HNT dispersion within PVA matrices. On the contrary, increasing the HNT content inevitably causes noticeable particle aggregation with more stress concentration sites around HNT agglomerates as a result of potential crack initiation to deteriorate the mechanical performance of bionanocomposites. With repect to PVA/Cloisite 30B clay bionanocomposites, their tensile strengths are increased by 18.8% and 28.4% with the incorporation of 3 and 5 wt % Cloisite 30B clays, respectively. This finding is ascribed to more uniform clay dispersion as well as the formation of stronger matrix–filler network structures, resulting from increasing hydrogen bonding between these constituents due to larger clay surface areas [47,48]. When the Cloisite 30B clay content increases up to 10 wt %, the tensile strength of PVA/Cloisite 30B clay bionanocomposites is decreased by 5.16% as opposed to that of near PVA. This phenomenon suggests that the aggregation of nanofillers at high clay content levels can undermine the tensile strengths of bionanocomposites. On the contrary, the tensile strengths of PVA/NBC bionanocomposites have the initial improvement up to 147.94 MPa (by a maximum level of 110.4%) when the NBC content increases from 0 to 3 wt %. Beyond 3 wt % NBCs, the tensile strengths of such bionanocomposites tend to decline until they reach the lowest strength levels of 96.34 MPa at the NBC content of 10 wt %. However, such lowest strength levels are still better than that of neat PVA. Overall, both tensile moduli and tensile strengths of PVA/NBC bionanocomposites are consistently superior to those of PVA/HNT bionanocomposites and PVA/Cloisite 30B bionanocomposites to confirm the most effective reinforcement efficiency of NBCs among all three different nanofillers.

The elongation at break and tensile toughness of PVA/Cloisite 30B clay bionanocomposites and PVA/NBC bionanocomposites were continuously decreased, especially beyond the nanofiller content of 3 wt %, as shown in Figure 7c,d. The maximum decreasing levels by approximately 59.5% and 58% in elongation at break have been detected for PVA bionanocomposites reinforced with 10 wt % Cloisite 30B clays and NBCs, respectively. This finding can be associated with the stiffening effect from filler reinforcements of NBCs and Cloisite 30B clays to restrict the mobility of PVA molecular chains, thus resulting in the overall flexibility reduction in bionanocomposite films [49]. As for PVA/HNT bionanocomposites, elongation at break and tensile toughness are increased by 12.7% and 16.9% with the incoproration of 3 wt % HNTs. Beyond that, they both remarkably diminish until maximum reductions of 50% and 45.3% take place at the HNT content of 10 wt %, respectively, as opposed to those of PVA. The former finding can be explained by good particle–matrix interactions with more uniform particle dispersion at low HNT contents. Whereas, the latter result can be associated with typical particle agglomeration at high HNT contents up to 10 wt % with the disapearance of the ‘nano effect’ of HNTs, since most HNT aggregates become less favourable microfillers with poor particle dispersion. As such, those HNT aggregates act as typical defects with high stress concentration prone to crack initiation towards mechanical failure, thus leading to poor material toughness [36].

In this study, the incorporation of three different nanofillers (i.e., HNTs, Cloisite 30B clays, and NBCs) has successfully enhanced the mechanical properties of PVA bionanocomposite films. According to our results, the highest increasing level among PVA/HNT bionanocomposites and PVA/NBC bionanocomposites can be achieved at the filler content of 3 wt %, as opposed to the optimum content of 5 wt % for PVA/Cloisite 30B clay bionanocomposites. Nonetheless, such an increasing rate achieved in PVA bionanocomposites using three types of nanofillers appears to be quite different, which is associated with various nanofiller features in terms of their structures and geometries, as well as the degree of compatibility between nanofillers and polymer matrices. With respect to nanofiller shape, it is well known that NBCs are regarded as 3D nanofillers as opposed to 2D nanofillers for HNTs and 1D nanofillers for Cloisite 30B clays. Different nanofiller shapes thereby influence the overall interfacial areas between fillers and polymer matrices, which plays a key role in the improvement of tensile strengths of nanocomposites with different levels of filler–matrix interactions. The second aspect is related to the structures, particularly the location of hydroxyl groups for nanofiller structures and amounts of hydroxyl groups within nanofillers. In the case of NBCs, hydroxyl groups are located inside their pores, which tend to more closely interact with PVA from a 3D point of view. As for HNTs, the majority of hydroxyl groups are constrained in inner tubes between layers, which makes the matrix–HNT interaction limited to the inner tubes of HNTs only. Moreover, in the case of Cloisite 30B clays, hydroxyl groups are located between layered structures, which means that the interactions between polymer matrices and platelet-like clays are limited to small highly constrained interlayer areas. The highest tensile moduli and tensile strength of bionanocomposite films have been achieved with the incorporation of NBCs relative to that of PVA. Several reasons can explain the above-mentioned results in relation to the mechanical properties of bionanocomposites. First, 3D nanofiller shape of NBCs can be generated at low nanofiller contents and in small particle sizes with relatively large interfacial areas, as compared with 2D HNTs and 1D Cloisite 30B clays. Liu and Brinson [2] investigated the effect of nanofiller geometry on the reinforcing efficiency of nanocomposites, which shows that at a low nanofiller content with the random nanofiller orientation, the transverse modulus of nanoparticle-based nanocomposites significantly exceeded those of nanotube-based nanocomposites, as well as nanoplatelet-based nanocomposites. Schadler et al. [50] reported that in the case of a nanocomposite system, with the incorporation of nanoparticles and nanotubes having a nanofiller diameter of 10 nm at the volume fraction of 10 vol%, the volume fraction of interfacial polymers was about 30% in the case of nanoparticle-based nanocomposites as opposed to only 10% for nanotube-based nanocomposites. The second reason in relation to the high mechanical performance of PVA/NBC bionanocomposites can be ascribed to the chemical structures of nanofillers in terms of the amounts and locations of hydroxyl groups in order to control the nanofiller dispersion within bionanocomposites, thus reflecting upon the bonding between polymer matrices and nanofillers. Pakzad et al. [51] indicated that the number and nature of hydrogen bonds had a substantial effect on the mechanical properties of nanocomposites. In the case of PVA/3 wt % NBC bionanocomposites, NBCs have highly porous structures with a large amount of hydroxyl groups located inside these pores when NBCs are uniformly dispersed. As confirmed by the FTIR and XRD results, polymeric chains enter these pores and form both hydrogen and mechanical bondings. Such two bonding types can be particularly recognised for NBCs as compared to Cloisite 30B clays and HNTs, thus significantly reflecting upon the enhanced mechanical properties of nanocomposites [4,5]. In case of PVA/5 wt % Cloisite 30B clay bionanocomposites in comparison to PVA/5 wt % HNT bionanocomposites, the strong adhesion of clays in polymer matrices associated with uniform clay dispersion leads to the strong interfacial bonding between nanoclays and polymer matrices, which thus significantly contributes to the improvement of mechanical properties of bionanocomposites.

The dispersion state of nanofillers can also influence the mechanical properties of PVA bionanocomposites. As mentioned earlier, NBCs have a better dispersion state than Cloisite 30B clays and HNTs. The incorporation of NBCs within PVA matrices yields smoother bionanocomposite films with higher tensile strength when compared with those of PVA/HNT bionanocomposites and PVA/Cloisite 30B clay bionanocomposites. The better dispersion state of NBCs improves their intercation with PVA matrices, thus leading to the higher tensile strengths of PVA/NBC bionanocomposites. On the contrary, increasing the nanofiller content appears to induce higher surface roughness as well as lower tensile strengths of nanocomposites, which indicates that nanofiller agglomeration apparently has detrimental effect on the improvement of tensile strength. This is particularly the case for PVA/HNT bionanocomposites due to the poor HNT dispersion state. On the contrary, PVA/3 wt % HNT bionanocomposites yield much higher elongation at break and fracture toughness as opposed to those of neat PVA, which are different from PVA/3 wt % NBC bionanocomposites and PVA/5 wt % Cloisite 30B clay bionanocomposites with corresponding lower values. Such results can be clearly explained by two major reasons. The first reason is ascribed to the number of nanoparticles depending on the volume and volume fraction of nanoparticles in bionanocomposites. At the same volume fraction, the number of NBCs is significantly larger than those of tubular HNTs or platelet-like Cloisite 30B clays. As such, this finding results in increasing the number of available reinforcements for improving the matrix rigidity and then decreasing the fracture toughness [43,52]. The second reason is related to the mechanism of fracture toughness, including the pre-crack effect for the fracture of nanocomposites. In general, crack deflection and crack pinning are most well-known mechanisms resulting in an increase in fracture energy [53], and consequently an increase in fracture toughness of nanocomposites. In both mechanisms mentioned earlier, the crack growth path can increase as long as those cracks reach nanofiller regions and the reinforcement shape highly affects the amount of crack deviation from their initial path. Since HNTs have larger lateral dimensions in comparison with NBCs, the cracks tend to pass over longer distances in PVA bionanocomposites reinforced with HNTs. Moreover, crack bridging is also well recognised as a fracture mechanism in nanocomposites reinforced with nanoparticles with a high aspect ratio [43]. An ideal situation in this mechanism occurs when nanotube fillers are still embedded in matrices while aligned in a perpendicular direction to crack faces. Consequently, PVA/HNT bionanocomposites achieve less reduction in fraction toughness when compared with the PVA/Cloisite 30B bionanocomposites and PVA/NBC bionanocomposites in Figure 7d.

### 3.7. Fracture Morphology

**Figure 8:**
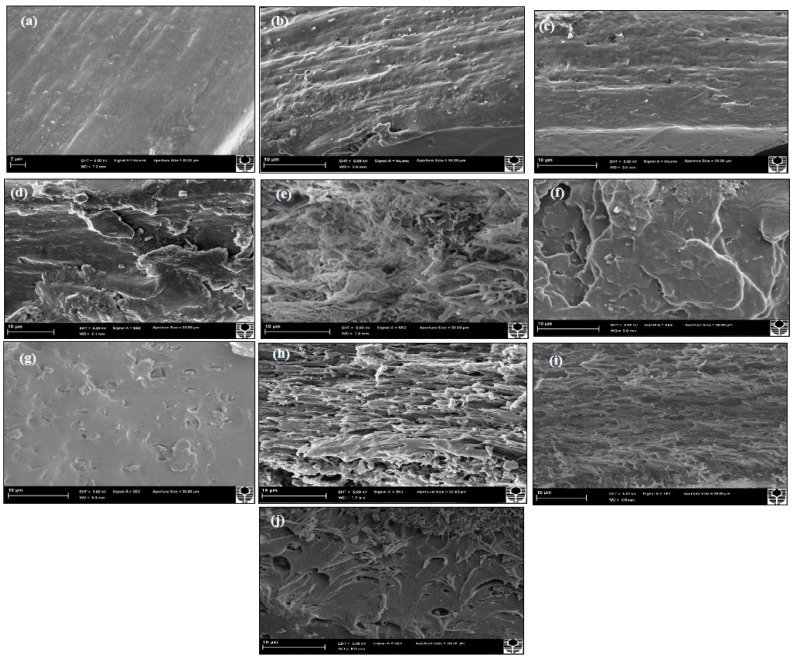
SEM micrographs of tensile fracture surfaces: (**a**) PVA, (**b**) PVA/3 wt % HNT bionanocomposites, (**c**) PVA/5 wt % HNT bionanocomposites, (**d**) PVA/10 wt % HNT bionanocomposites, (**e**) PVA/3 wt % Cloisite 30B clay bionanocomposites, (**f**) PVA/5 wt % Cloisite 30B bionanocomposites, (**g**) PVA/10 wt % Cloisite 30B clay bionanocomposites, (**h**) PVA/3 wt % NBC bionanocomposites, (**i**) PVA/5 wt % NBC bionanocomposites and (**j**) PVA/10 wt % NBC bionanocomposites. Note that Figure 8a shows the SEM micrograph with a scale bar of 2 µm while the rest of micrographs are labelled with a scale bar of 10 µm.

Figure 8 shows typical SEM micrographs of cross-sectional fracture surfaces for PVA, PVA/HNT bionanocomposites, PVA/Cloisite 30B clay bionanocomposites, and PVA/NBC bionanocomposites. It can be clearly seen in Figure 8b,e that PVA bionanocomposites reinforced with 3 wt % of HNTs and Cloisite 30B clays reveal much rougher fractured surfaces when compared with that of neat PVA films, as illustrated in Figure 8a. Moreover, 3 wt % HNTs or Cloisite 30B clays are distributed uniformly within PVA matrices. The good dispersion of both nanoparticles and the strong interaction between clay particles and polymer matrices clearly contribute to the reinforcing effect, as reflected by the increase in both tensile strength and elastic modulus. Nevertheless, in both PVA/HNT bionanocomposite and PVA/Cloisite 30B clay bionanocomposite systems, uniform multi-layered structures have not been achieved similar to those detected in PVA/3 wt % NBC bionanocomposites, as illustrated in Figure 8h. Such results are indicative of high NBC dispersability as compared with those of HNTs and Cloisite 30B clays, resulting in the highest mechanical performance. Meanwhile, at the HNT content of 5 wt %, particle–particle interactions are more favourable than their particle–matrix counterparts, as evidenced by more filler agglomeration in the presence of debonding and microvoid effects depicted in Figure 8c. Such defects in nanocomposite systems give rise to the decreasing tensile strengths of PVA/HNT bionanocomposites. However, as for PVA/5 wt % Cloisite 30B clay bionanocomposites, the clay dispersion appears to be still relatively uniform with the presence of small particle agglomeration shown in Figure 8f. With increasing the nanofiller contents of HNTs and Cloisite 30B clays from 5 to 10 wt %, the fracture surfaces of bionanocomposites films are altered from ductile characteristic to more brittle behaviour, as illustrated in Figure 8d,g, respectively. Similar phenomena are also found in PVA/NBC bionanocomposites according to Figure 8j. As is well known, decreasing surface roughness reveals that the failure mode of PVA bionanocomposite films can be quite different by changing from ductile to brittle fracture [54], which is consistent with the reduced mechanical properties of bionanocomposite films in this study.

### 3.8. Thermal Properties

PVA is a water-soluble semicrystalline polymer, in which high physical interchain and intrachain interactions exist because of the typical hydrogen bonding between hydroxyl groups. The inclusion of nanoclays with hydroxyl groups can alter the intramolecular and intermolecular interactions of PVA molecular chains. This may affect both the crystallisation behaviour and physical structures of PVA. Similar observations can be found in previous studies dealing with PVA/HNT bionanocomposites [46,55].

**Figure 9:**
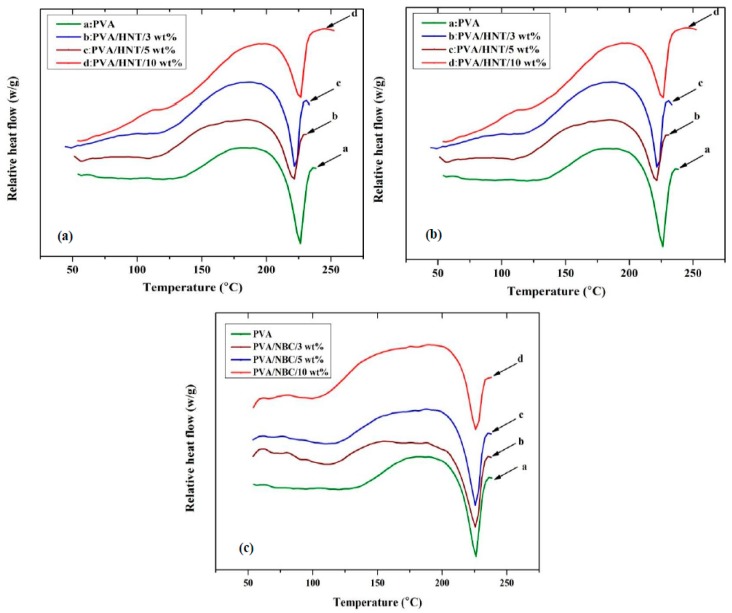
Differential scanning calorimetry (DSC) thermograms of PVA bionanocomposites reinforced with (**a**) HNTs, (**b**) Cloisite 30B clays, and (**c**) NBCs. The curves are shifted vertically for clarity.

Figure 9 shows the DSC results of PVA*/*HNT bionanocomposites, PVA/Cloisite 30B clay bionanocomposites, and PVA*/*NBC bionanocomposites. The summarised data of these thermal characteristics are reported in Table S1 in Supporting Information. For PVA*/*HNT bionanocomposites, it is clearly seen that the glass transition temperature *T_g_* of PVA becomes unchanged with the addition of HNTs in bionanocomposite films, implying that HNTs do not play an important role in inhibiting the chain mobility of PVA molecules. Qiu and Netravali [46] also reported a similar result in *T_g_* with the incorporation of HNTs into PVA. Such a finding might be related to the reduction in the entanglements and interactions of PLA polymeric chains with HNT inclusions. The relatively unchanged *T_g_* in PVA/HNT bionanocomposites, as compared to that of neat PVA, may arise from different nanofiller geometries. The diameters of HNTs are at a nanoscaled level as opposed to submicron- or microsized tubular lengths that considerably exceed the typical gyration radii of polymeric chains [4]. As a result, HNTs cannot be completely wrapped by PVA molecular chains leading to many voids surrounding HNT particles. On the contrary, the high *T_g_* values for all PVA/Cloisite 30B clay bionanocomposites are evident, as opposed to that of neat PVA. With the incorporation of 3, 5 and 10 wt % Cloisite 30B clays, the *T_g_* values of such PVA bionanocomposites are moderately enhanced up to 67.5, 70.2 and 71.8 °C, respectively when compared with that of neat PVA at 65.19 °C. This phenomenon can be attributed to the confinement of polymeric chains by intercalated clay structures to prevent their segmental motions [1], which has also been recorded in PVA/MMT nanocomposites [56,57], PVA/bentonite nanocomposites [58], as well as PVA/starch/MMT nanocomposites [59]. In the case of PVA/NBC bionanocomposites, the *T_g_* increases monotonically up to 75.06 °C with increasing the NBC content from 0 to 10 wt % accordingly. The incoporation of rigid NBC particles can restrict the chain mobility of PVA matrices so that higher *T_g_* values are required for the phase transformation of nanocomposites from a glassy state to a rubbery state. This finding is well known for many types of nanofillers such as nanoclays, GOs, CNTs, HNTs, etc. [1]. Overall, the *T_g_* values of PVA bionanocomposite films with the incorporation of NBCs and Cloisite 30B clays are much higher than those of PVA/HNT bionanocomposites. Such results indicate that NBCs and Cloisite 30B clays can restrict PVA chains more efficiently, as evidenced by the enhanced mechanical properties of corresponding bionanocomposite films. According to previous studies [60,61], the phenomenon of increasing *T_g_* is primarily associated with the reduction in polymeric chain mobility by incorporating inorganic nanofillers. The incorporation of nanoparticles into polymer matrices can change the distribution of chain segments, which is most likely due to a change in the chain packing density in the vicinity of nanofiller surfaces. It should be noted that filler geometry may play a critical role to influence *T*_g_. NBCs and Cloisite 30B clays have different nanofiller shapes to render the absorption of polymeric chains with entangled structures on their surfaces when nanofiller diameters are comparable to the gyration radii of polymeric chains. As such, it leads to increasing the packing density for polymeric chains and restricting their chain mobility as a result of higher *T*_g_ values. However, the incorporation of HNTs into PVA matrices has a minor impact on increasing *T*_g_ instead, which is consistent with the previous work [55]. Although the diameter of HNTs is on the nanometer scale, their length turns to be submicron- or microsized, which becomes considerably higher than the typical gyration radii of polymeric chains. As a consequence, it is very difficult for polymeric chains to cover entire HNT structures. Moreover, the presence of microvoids along HNT lengths could offer free sites for the segments of polymeric chains, resulting in an insignificant increase of *T*_g_ [55].

The degree of crystallinity (χ_c_) of PVA slightly increases from 36.65% for neat PVA to 38.2% and 37.2%, 40% for corresponding bionanocomposites with the incorporation of 5 wt % HNTs and Cloisite 30B clays and 10 wt % of NBCs, respectively. This suggests that such nanofillers have minor effect on the crystalline phases of PVA matrices in bionanocomposites. On the other hand, the melting temperature *T_m_* of PVA bionanocomposites virtually has no change with the addition of Cloisite 30B clays and NBCs, as evidenced by the given *T_m_* ranges of 220.44–221.62 °C and 221–225 °C, respectively when compared with that of neat PVA at 222.91 °C. However, PVA/HNT bionanocomposites possess a moderate increase in *T_m_* up to 226.67 °C with the inclusion of 10 wt % HNTs. A similar phenomenon has also been noticed in PHBV/HNT nanocomposites [28] with their *T_m_* values being increased from 169 to 173 °C when incoprorated with 5 wt % HNTs. Based on their XRD results, thicker and more oriented HNT/PHBV structures could be formed, leading to higher melting temperatures.

**Figure 10:**
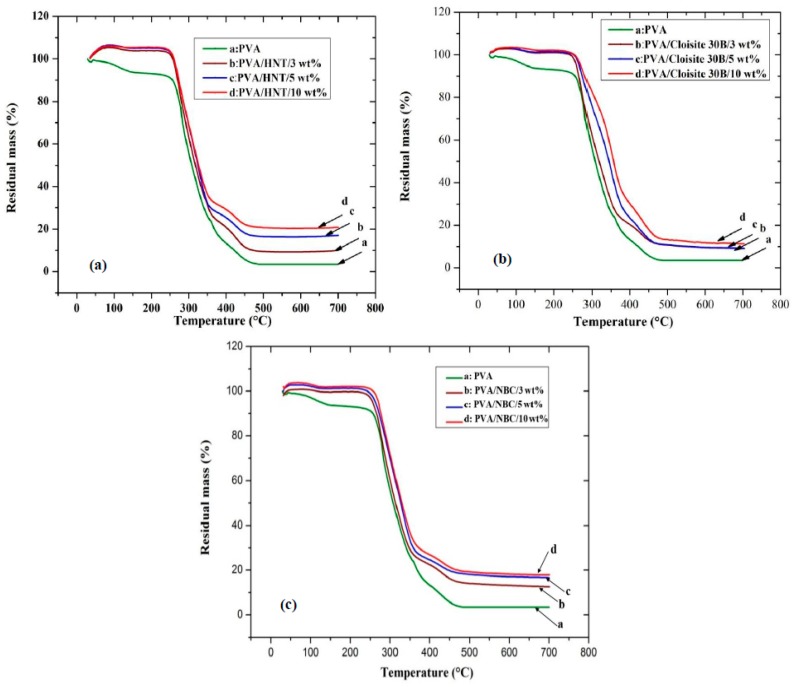
TGA curves for PVA bionanocomposites reinforced with (**a**) HNTs, (**b**) Cloisite 30B clays, and (**c**) NBCs.

**Figure 11:**
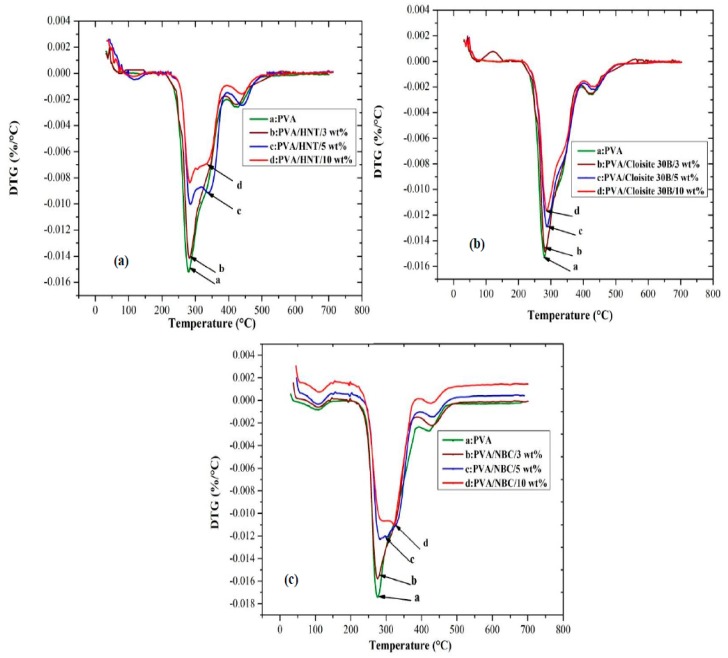
DTG curves for PVA bionanocomposites reinforced with (**a**) HNTs, (**b**) Cloisite 30B clays, and (**c**) NBCs.

The thermal decomposition behaviours of PVA/HNT bionanocomposites, PVA/Cloisite 30B clay bionanocomposites, and PVA/NBC bionanocomposites have been evaluated using thermogravimetric analysis (TGA) with the corresponding results being presented in Figure 10 and Figure 11, as well as Table S1 in Supporting Information. The relevant results for both systems reveal the existence of three major degradation steps according to previous studies [21]. Initially, the first degradation takes place at 107 °C owing to the breakage of hydrogen bonds, impurities, and monomers of vinyl alcohol. Then, the second degradation occurring at 274 °C involves a dehydration reaction on PVA molecular chains, the degradation of main backbones, as well as the decomposition of organic clays. This process is accompanied by a drastic mass change caused by the removal of organic compounds such as CO_2_ and the long molecular chains of alkyl derivatives. Finally, the third degradation step appears at a temperature level below 429 °C with more complexity including the further degradation of polyene residues to yield the carbon and hydrocarbon. The incorporation of HNTs, Cloisite 30B clays, and NBCs can increase the thermal stability of PVA by reducing the weight loss and increasing the decomposition temperatures, as presented in Figure 10 and Figure 11. As for PVA/HNT bionanocomposites, the decomposition temperature at 5% weight loss *T_5%_* increases from 200.2 °C for PVA to 265.3, 268.1, and 270.2 °C for PVA bionanocomposites reinforced with 3, 5 and 10 wt % HNTs, respectively. Such a finding suggests that HNTs work as an effective barrier material to heat and mass transfer. Moreover, the intrinsic hollow tubular structures of HNTs can produce the traps for volatile particles, thus improving the thermal stability by delaying the mass transfer during a decomposition process. Moreover, as clearly seen from the derivative thermogravimetry (DTG) curves in Figure 11a, the maximum decomposition temperature *T_d_* of PVA shifts to a higher temperature level, which means that the dehydration process is hindered, resulting from strong interactions between PVA matrices and HNTs, as well as an important role of HNTs as good barrier materials to increase the thermal resistance of PVA bionanocomposites. Furthermore, the second DTG peaks of PVA/HNT bionanocomposites reinforced with 5 and 10 wt % HNTs are much wider than that of neat PVA in the presence of main and side peaks as compared to the single DTG peak for PVA at the same step. This signifies that a single peak for PVA can be attributed to the eliminated reaction while the side and main peaks of PVA/HNT bionanocomposites correspond to the eliminated reaction as well as the overlap of continual elimination and chain-scission reaction with the requirement of more energy to accrue at high temperatures [60].

On the other hand, in the case of PVA/Cloisite 30B clay bionanocomposites, with increasing the clay content, the thermal stability of bionanocomposites improves as compared to that of PVA, which is evidently demonstrated from consistently high *T_5%_*, the decomposition temperature at 80% weight loss *T_80%_*, and *T_d_* values shown in Figure 10b. For instance, the *T_80%_* of PVA increases from 363.5 to 407 °C with the inclusion of 5 wt % Cloisite 30B clays. Such a result is in good agreement with previous studies of PVA/MMT nanocomposites [21,62]. Moreover, the shift in the decomposition temperatures *T_d_* for PVA/Cloisite 30B bionanocomposites depicted in Figure 11b suggests the hindrance of a dehydration process. Such a finding in thermal stability is associated with the presence of nanolayers acting as the barriers to maximise the heat insulation and minimise the permeability of volatile degradation products in the materials. This increase is also attributed to the decrease in oxygen permeability related to good clay dispersion in PVA matrices.

Moreover, the thermal stabilities of PVA/NBC bionanocomposites are improved significantly relative to that of PVA, as evidenced by consistently higher *T_5%_*, *T_80%_*, and *T_d_* values, as shown in Figure 10c. The degree of thermal stability of bionanocomposites is even more pronounced when incorporated with NBCs in relation to *T_5%_* and *T_80%_*. The *T_5%_* of PVA/3 wt % NBC bionanocomposites was determined to be 256.3 °C, and it increases to 262.95 °C with the incorporation of 5 wt % NBCs, which is relatively similar to that of PVA/5 wt % Cloisite 30B bionanocomposites at 261.1 °C. On the other hand, the *T_5%_* of PVA/3 wt % HNT bionanocomposites appears to be determined at 265.29 °C, which is significantly higher as compared with PVA bionanocomposites reinforced with NBCs and Cloisite 30B clays. On the contrary, the *T_80%_* of PVA/3 wt % NBC bionanocomposites has been found to be 390.67 °C and reaches 440.28 °C with the inclusion of 5 wt % NBCs, which is significantly higher than those of PVA bionanocomposites reinforced with HNTs and Cloisite 30B clays. This result infers that the maximum thermal stability is achieved in the presence of NBCs as compared with HNTs and Cloisite 30B clays. As opposed to other nanofillers, the better NBC dispersion within PVA matrices takes place along with the higher barrier towards the thermal degradation. Therefore, such a barrier effect can counterbalance the degradation drawback with the further improvement of thermal stability [5].

The shift of maximum decomposition temperatures for the first and second degradation steps *T_d_* and *T_d_’* in Figure 11c also means the obstruction of the dehydration process, which can result from the interaction between the hydroxyl groups of PVA and the hydroxyl groups on NBCs, as confirmed from our previous FTIR results. Furthermore, the mass loss process occurring in the second DTG peaks suggests that the thermal decomposition of PVA bionancomposites requires more reaction activation energy with the higher reaction order [63]. This finding may be attributed to the existence of NBCs working as effective barrier materials to limit the exothermicity of pyrolysis reaction with the better thermal resistance of PVA bionanocomposites. The wider DTG peaks of PVA/NBC bionanocomposites beyond 3 wt % NBCs at the second decomposition step demonstrates a similar trend to those of the corresponding PVA/HNT bionanocomposites along with the same dual-peak effect, as mentioned elsewhere [63].

## 4. Conclusions

PVA bionanocomposites reinforced with Cloisite 30B clays, HNTs, and NBCs have been successfully prepared and characterised. The following conclusions can be drawn.

The properties of PVA/HNT bionanocomposites are remarkably affected when embedding HNTs, which primarily depend on the HNT content. Nanofiller dispersion can lead to various morphological structures resulting in enhanced mechanical properties at different levels for such bionanocomposites. In particular, the incorporation of 3 wt % HNTs has improved mechanical properties, while increasing the HNT content beyond that causes the decreases in tensile strength, elongation at break, and tensile toughness of PVA/HNT bionanocomposites, which is possibly associated with the typical filler–matrix debonding effect. Moreover, the thermal properties of PVA/HNT bionanocomposites in terms of the degree of crystallinity, melting temperature, and thermal stability are enhanced with increasing the HNT content as opposed to those of neat PVA films.

The morphological structures of PVA/Cloisite 30B clay bionanocomposites demonstrate uniform clay dispersion within PVA matrices in combined clay exfoliated and intercalated structures, which are in agreement with those obtained from XRD results. The tensile strengths and Young’s moduli of PVA/Cloisite 30B clay bionanocomposite films are increased considerably with increasing the clay content up to 5 wt % amid decreasing elongation at break and fracture toughness. The thermal properties of PVA/Cloisite 30B clay bionanocomposites are improved compared to those of neat PVA films due to the strong hydrogen-bonding interactions between PVA matrices and nanofillers.

The effect of different nanofiller shapes and structures on the properties of PVA/NBC bionanocomposite films reveals that the maximum tensile strength and tensile modulus can be achieved with the incorporation of NBCs. This can be related to the large amount of interphase resulting from a high degree of filler dispersion in the case of PVA/NBC bionanocomposites relative to those reinforced with HNTs and Cloisite 30B clays. Moreover, the thermal stability of PVA/NBC bionanocomposites is remarkably enhanced with the inclusion of NBCs in contrast to those incorporated with HNTs and Cloisite 30B clays. This can be ascribed to the uniform dispersion of NBCs to generate more efficient interfacial regions as opposed to other nanofillers.

## Supplementary Materials

The following are available online at https://www.mdpi.com/2073-4360/12/2/264/s1, Figure S1: Characterisations of PVA/ 3wt% Cloisite 30B clay bionanocomposites: (a) height mapping image and (b) height profiles on cut-line sections A_S1_-B_S1_ and A_S2_-B_S2_, Figure S2: Frequency distributions of dimensions of HNTs embedded within PVA/HNT bionanocomposites at different HNT contents: (a) and (b) for HNT length and diameter (3 wt% HNTs), (c) and (d) for HNT length and diameter (5 wt% HNTs), as well as (e) and (f) for HNT length and diameter (10 wt% HNTs), Figure S3: Frequency distributions of dimensions of Cloisite 30B clays embedded within PVA/Cloisite 30B clay bionanocomposites at different clay contents: (a) and (b) for clay length and thickness (3 wt% Cloisite 30B clays), (c) and (d) for clay length and thickness (5 wt% Cloisite 30B clays), as well as (e) and (f) for length and thickness (10 wt% Cloisite 30B clays), Figure S4: Frequency distributions of dimensions of NBCs embedded within PVA/NBC bionanocomposites at different NBC contents: (a) and (b) for NBC thickness and diameter (3 wt% NBCs), (c) and (d) for NBC thickness and diameter (5 wt% NBCs), as well as (e) and (f) for NBC thickness and diameter (10 wt% NBCs), Table S1: Thermal properties of PVA bionanocomposite films.
