# Supplementary material for: The Role of Nanoparticle Shapes and Structures in Material Characterisation of Polyvinyl Alcohol (PVA) Bionanocomposite Films
Source: Polymers (Basel). 2020 Jan 25;12(2):264. doi: 10.3390/polym12020264 (PMC7077314; doi:10.3390/polym12020264)
Supplement: Supplementary file 1 [file polymers-12-00264-s001.pdf]

Supporting Information

The Role of Nanoparticle Shapes and Structures in Material Characterisation of Polyvinyl Alcohol (PVA) Bionanocomposite Films

Mohanad Mousa [1,2] and Yu Dong [1,*]

[1] School of Civil and Mechanical Engineering, Curtin University, GPO Box U1987, Perth 6845, Australia; mohanadmousa616@yahoo.com

[2] Shatrah Technical Institute, Southern Technical University, Basra 61001, Iraq

* Correspondence: Y.Dong@curtin.edu.au; Tel.: +61-8-9266-9055

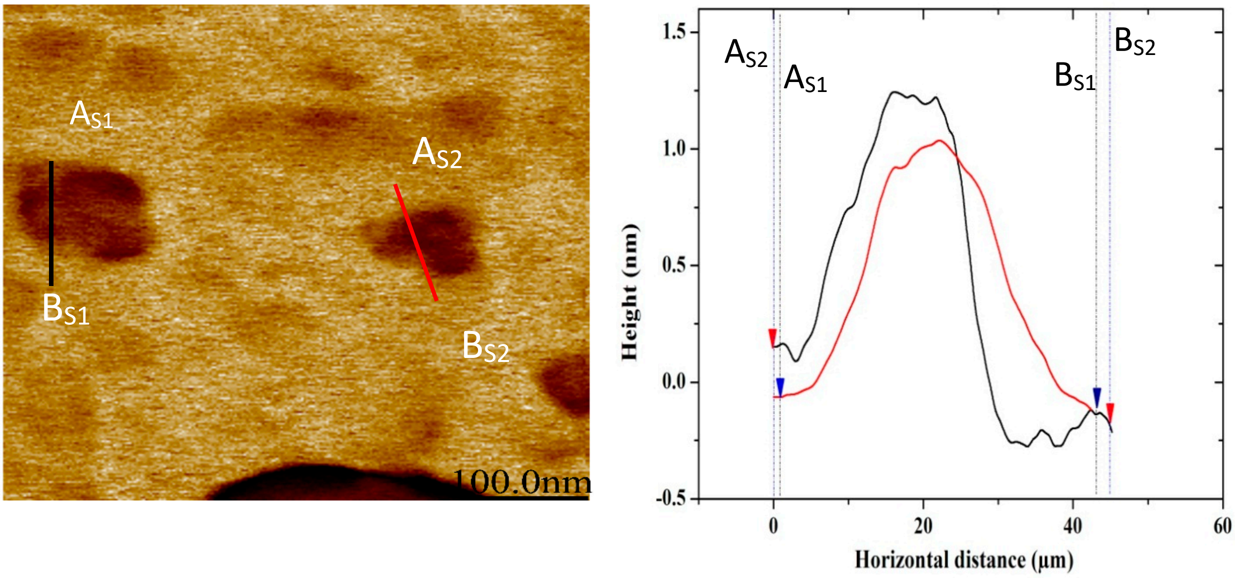

(a) (b)

Figure S1. Characterisations of PVA/ 3wt% Cloisite 30B clay bionanocomposites: (a) height mapping image and (b) height profiles on cut-line sections A_{S1}-B_{S1} and A_{S2}-B_{S2}.

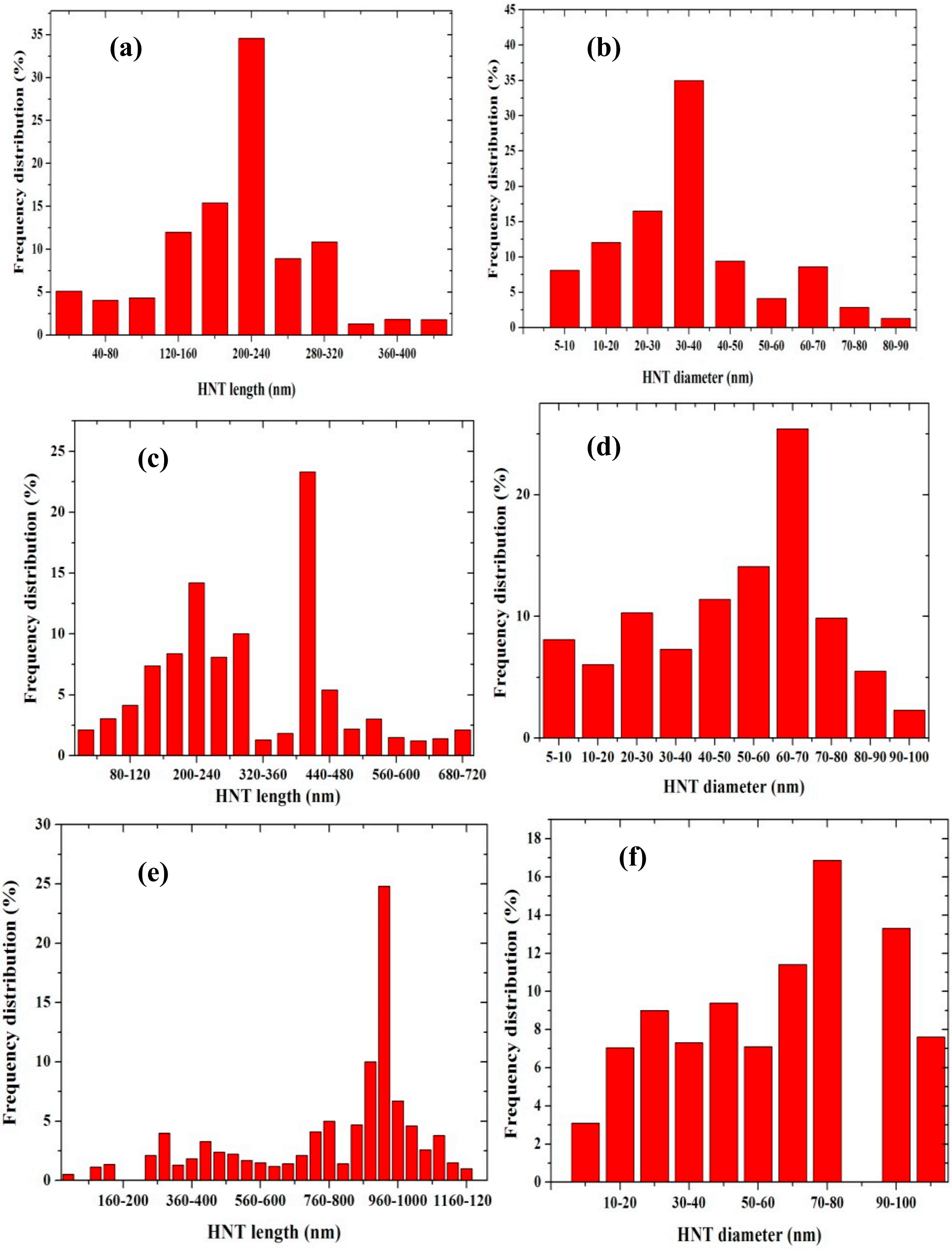

Figure S2. Frequency distributions of dimensions of HNTs embedded within PVA/HNT bionanocomposites at different HNT contents: (a) and (b) for HNT length and diameter (3 wt% HNTs), (c) and (d) for HNT length and diameter (5 wt% HNTs), as well as (e) and (f) for HNT length and diameter (10 wt% HNTs).

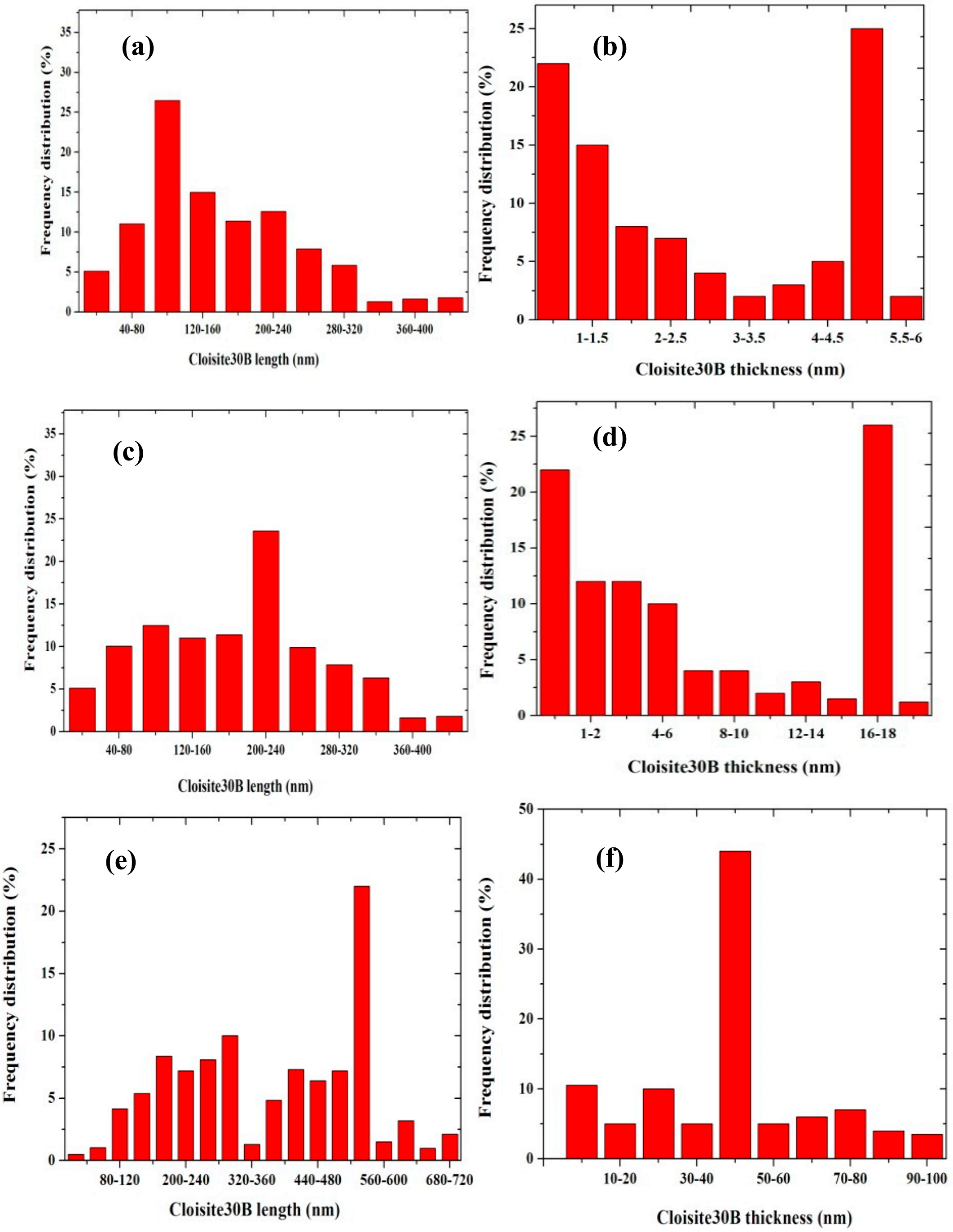

Figure S3. Frequency distributions of dimensions of Cloisite 30B clays embedded within PVA/Cloisite 30B clay bionanocomposites at different clay contents: (a) and (b) for clay length and thickness (3 wt% Cloisite 30B clays), (c) and (d) for clay length and thickness (5 wt% Cloisite 30B clays), as well as (e) and (f) for length and thickness (10 wt% Cloisite 30B clays).

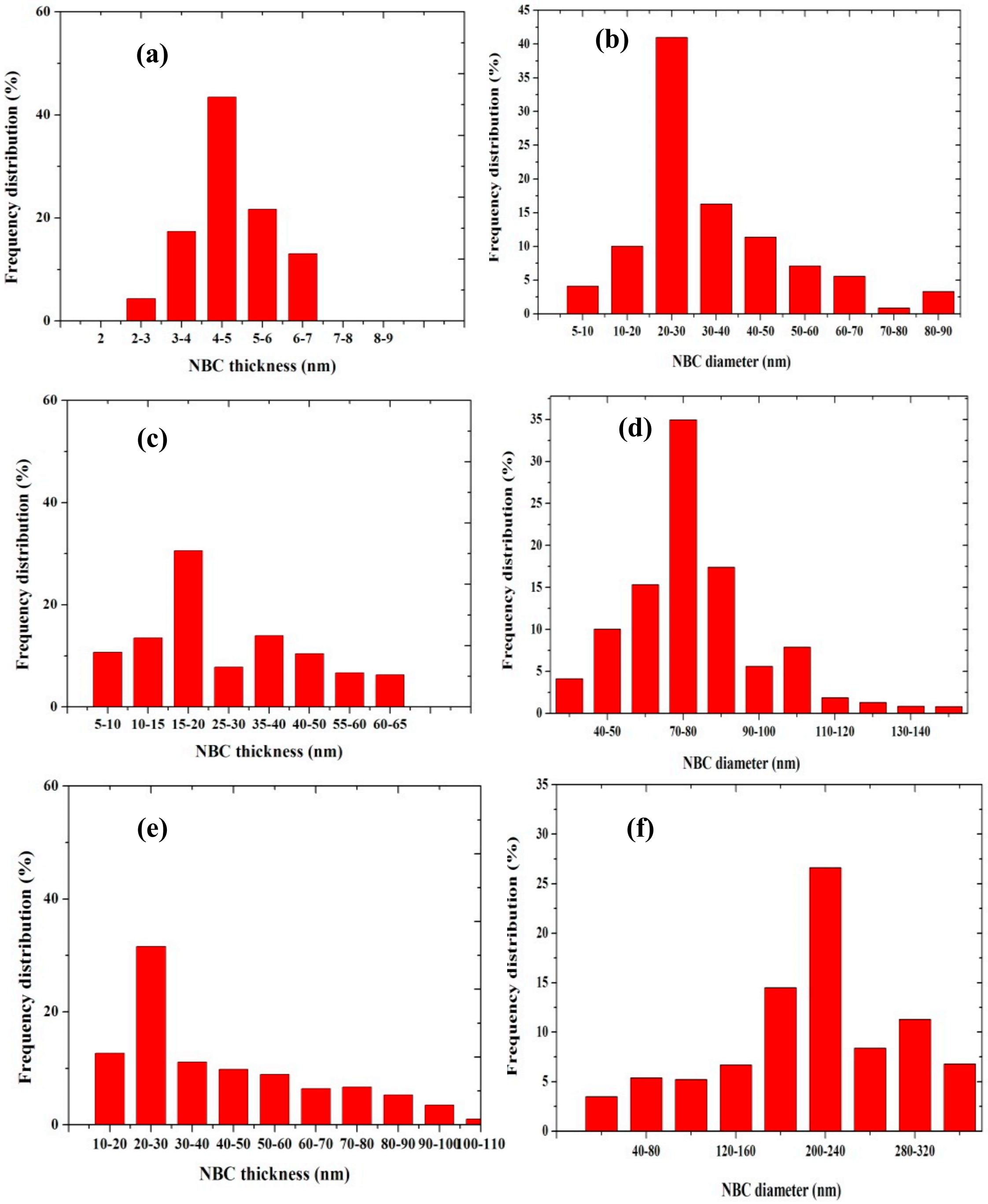

Figure S4. Frequency distributions of dimensions of NBCs embedded within PVA/NBC bionanocomposites at different NBC contents: (a) and (b) for NBC thickness and diameter (3 wt% NBCs), (c) and (d) for NBC thickness and diameter (5 wt% NBCs), as well as (e) and (f) for NBC thickness and diameter (10 wt% NBCs).

Table S1. Thermal properties of PVA bionanocomposite films.

Material type	T_g (°C)	ΔH_m (J/g)	χ_c (%)	T_m (°C)	$T_{5\%}$ (°C)	$T_{80\%}$ (°C)	T_d (°C)	T_d' (°C)
PVA	65.19	50.8	36.65	222.91	200.15	363.5	274.23	
PVA/HNT/3 wt%	65.13	50.5	37.6	224.74	265.29	387	283.2	
PVA/HNT/5 wt%	65.86	50.2	38.2	225.13	268.06	422.87	286.47	340.12
PVA/HNT/10 wt%	64.91	44.15	35.4	226.67	270.21	428.03	287.13	335.26
PVA/Cloisite 30B /3 wt%	67.5	49.6	36.9	220.44	253.3	387.9	282.21	
PVA/ Cloisite30B /5 wt%	70.2	49.09	37.2	221.62	261.1	407.12	287.41	
PVA/ Cloisite30B /10 wt%	71.8	45.01	36.1	221.06	265.3	435.3	290.13	
PVA/NBC/3 wt%	70.53	50.01	37.21	222.12	256.3	390.67	278.48	
PVA/ NBC/5 wt%	73.46	49.77	38.83	221.57	262.96	440.28	283.4	303.77
PVA/ NBC/10 wt%	75.06	48.16	40	222.63	270.73	464.03	294.61	324.12
